# The War and Professional Prospects

**Published:** 1914-09-19

**Authors:** 


### THE WAR AND PROFESSIONAL PROSPECTS.

### The Outlook for Education and Practice.

Parents who have been considering the possibilities of a medical career for their sons, and young people of both sexes who have been making up their minds as to whether they shall enrol their names as medical students at the beginning of the coming session, have inevitably found occasion to pause in their plans, owing to the unforeseen events of the past month. And one result of these fresh deliberations seems likely to be that a certain number will draw back at this, the eleventh hour, deciding either to temporise until the Easter term, or to give up altogether the idea of becoming medical practitioners. It is, indeed, a very serious thing for the average family man to launch a son on a career entailing constant expenditure for at least five years, just as the country is embroiled in a struggle the end of which no man can foresee, and the initial consequences of which have already incurred such a disturbance of finance as has not been previously seen in this generation. The medical curriculum is both exacting and expensive, whilst even the attainment of a qualification is no sure guarantee of immediate remunerative employment—except, be it noted, at such a time as the present, when there is an exceptional demand for doctors for military service. As it takes at least five years to obtain the necessary licence to practise, it is obvious that the greatest burden will have to be borne during just that coming period when everyone will be trying to economise as far as possible. Moreover, the demand for men to create the new armies about to be raised will undoubtedly absorb a very considerable proportion of that class of young men from whom the medical schools are accustomed to derive chief support.

With so many possibilities of employment in the ranks, as officers, or under the Red Cross, the civil hospitals will by comparison lose much of their powers of attraction. Military duties of any kind possess an irresistible glamour for boys fresh from school or in their early college years; and it is difficult to find fault with the spirit that in time of war leads our youth to find occupation under the banner of Mars rather than under the mantle of Hippocrates. Thus in many homes just now the wishes of martial youth will attune with the doubts of a father who is naturally chary of facing additional expense of any kind, with the result that there will probably be many cancellations of intended registration.

All who have to do with the medical schools know that it is a very difficult thing to forecast the entry in any year, but there is certainly reason to suppose that the numbers of students joining the hospitals at the beginning of the ensuing session will fall notably below those of recent years. On first consideration it might appear that under such circumstances the prospects for those who do take up the study of medicine next month will be particularly rosy in that, not only throughout their students' career, but at the end of it, competition will be reduced to a minimum. But as a matter of fact, if there is lessened competition it will prove a very serious stumbling-block to the progress of medicine and surgery, in that it is just that excellent spirit of friendly rivalry pervading all our scientific institutions that has led to their attainment of a high standard of excellence. Smooth seas never make a good sailor, and too much easing of the path to qualification or academic distinction will certainly not turn out the best men. Further, there is an evident danger lest the standard of professional education may be lowered by the absence from the medical schools of many of those teachers whose names are known throughout the world of scientific medicine, and whose places must perforce be filled for the time being by junior men, whose only fault and handicap must be their lack of experience. In regard to these things the determining factor will be, of course, the duration of the war. A contest extending over several years would disastrously obstruct the progress of scientific medicine and research, so that those who have to pursue their studies in the shadow of the war-cloud may easily be deprived of advantages familiar to those who have immediately preceded them. That they may be compensated at the end for deprivation of scientific advantages by increased business opportunities is possible, owing to the dearth of doctors that a long war must occasion. But to the keen man it would be but a poor compensation, for medicine is not only a trade but an art, and to the keen student there can be no real compensation for the loss of guidance at the hands of such eminent teachers as those who have responded to the bugle-call, and who may not be seen in clinical theatre, laboratory, or ward until the sword is sheathed once more. From the educational point of view, then, it seems inevitable that a prolonged European struggle must result in the most regrettable disadvantages to professional prospects. Yet, on the other hand, the termination of the war within a matter of months would obviate this, and permit the intending student to reap to the fullest extent various opportunities that it appears will very likely occur as the result of present conditions, and which, indeed, are bound to present themselves, always provided peace be restored comparatively soon. It will be interesting to review the possibilities thus offered.

Assuming that the war will be concluded within a year it may be anticipated that at the end educational facilities will be practically as good as ever they have been, and that students entering the medical schools next month will miss the services of our best men only during the first year, which indeed is chiefly given up to pure science, and thus from this point of view may suffer very little disadvantage. At the same time, it is evident that a large number of doctors who have now accepted appointments under the War Office and Admiralty will remain in that employ in one capacity or another, so that for the next few years there should be greater opportunities of obtaining good appointments than has been the case lately. One result of this must be that public institutions, particularly hospitals, infirmaries, and asylums, will have to offer increased salaries in order to secure the services of well-qualified men; and, moreover, may probably have to afford rather more speedily than has hitherto appeared probable those facilities for married practitioners which are so much to be desired, particularly in regard to senior medical officers.

### Possible Opportunities.

If this prove correct, those who decide to rest their chances on the possibility of war being soon over, and take up work with the coming session, will in all probability, if their surmise be correct, find themselves in a position to obtain better emoluments in interesting public work than have been open to their predecessors. It is only natural that the possibilities offered by qualification should be first considered, but students should bear in mind the equally important fact that there will be exceptional opportunities during the next two or three years of obtaining scholarships, resident appointments and other numerous advantages of hospital life that usually fall to the lot of comparatively few fortunate men each year. The fact that the London hospitals have been for so long able to secure a constant succession of unsalaried house-physicians and house-surgeons bears witness to the remarkable competition there is for these appointments. Undoubtedly many keen men have to embark upon practice without being able to hold one of these posts owing to the fact that they cannot afford the time to compete more than once.

The possibility of such disappointments will obviously be minimised if the supply of students falls as low as may be expected now. Moreover the opportunities for assisting in research, performing minor operations, carrying out responsible clinical duties, and generally assuming a more important rôle in hospital work, are likely to be greater than they have ever been before; the only danger being, as has already been pointed out, that a long war may so denude the medical schools of their most experienced teachers, and lead to such a lack of medical students, that junior men will find the path far too easy, and attain distinctions without having necessarily gone through that slower and more systematic training which is essential for the turning out of a really first-class man. Should this happen, and students be able to obtain responsible posts with very little difficulty, the already high standard of medical attainment must be lowered and the scientific prospects of the professional man suffer in proportion.

Turning now to the effects that the war may be reasonably supposed to produce in regard to private medical work, it would appear that owing to the evident absorption of doctors into military service, with a consequently increased demand for candidates to fill well-paid posts in the hospitals, poor-law institutions, asylums and public health departments, there will be increased openings for private practitioners. Even before the war broke out it began to appear difficult to know where all the practitioners were to come from to fill the requirements of public offices and private practice. Now it is generally admitted that at the present time general practice does not appear promising to the keen young practitioner, who feels much rather inclined to take up public work of some kind, with the certainty of regular income and possibly a pension, than to embark on the uncertainties and irregularities of private medical work. Again, for the best men the prospects of private work were by no means increased by the institution of the panel. So that it has been evident recently to those who are accustomed to study these problems that the time might not be far distant when the supply of private doctors would scarcely equal the demand.

Hence with practitioners and students being absorbed in large numbers in an entirely unexpected direction, the outlook from the public point of view would appear to be worse than ever. Fewer doctors means on the one hand increased panel lists, and, on the other, increased fees amongst the more well-to-do classes; and this, of course, means larger incomes for those who decide to risk the perils of private work in years to come. It is certainly true that up to the present doctors have by no means had the best of discussions that have arisen in regard to their work and future prospects, but it does appear that now they may come into a very strong position, in that one of the problems of the near future may be a dearth of medical attendants in private life. And here again it would appear to be inevitable that one result of the war, whether it be short or long, must be very great opportunities for keen medical men as general practitioners. One advantage they will probably have over their predecessors is likely to be that, whilst the latter have found it difficult to persuade the lay public to rely on modern scientific methods of diagnosis and treatment as fully as would have been desirable, the doctor of the immediate future will, by virtue of lessened competition and strength of position, be able to insist more strongly on the use of such measures. Of these, one may mention modern methods of blood examination, inoculation treatment, *x*-ray diagnosis, exploratory operation, cardiology, and physiological chemistry. In regard to these things even the more educated members of the public are inclined to be very conservative and sceptical, and there is not the slightest doubt that much ill-health could be prevented, and a good many lives saved each year, if patients would give the general practitioners a freer hand in these matters. It should be one of the opportunities afforded to students now entering, when they have reached qualification, to insist on their private patients safeguarding themselves by the systematic use of scientific methods.

